# The role of impact on the meaning of generic sentences

**DOI:** 10.3389/fpsyg.2024.1363390

**Published:** 2024-09-23

**Authors:** Patricia Mirabile, Robert van Rooij, Katrin Schulz

**Affiliations:** Institute for Logic, Language and Computation (ILLC), University of Amsterdam, Amsterdam, Netherlands

**Keywords:** generics, experimental psychology, semantics, Bayesian methods, impact

## Abstract

Generic sentences (e.g., “Dogs bark”) express generalizations about groups or individuals. Accounting for the meaning of generic sentences has been proven challenging, and there is still a very lively debate about which factors matter for whether or not we a willing to endorse a particular generic sentence. In this paper we study the effect of *impact* on the assertability of generic sentences, where impact refers to the dangerousity of the property the generic is ascribing to a group or individual. We run three preregistered experiments, testing assertability and endorsement of novel generic sentences with visual and textual stimuli. Employing Bayesian statistics we found that impact influences the assertability, and endorsement, of generic statements. However, we observed that the size of the effect impact value may have been previously overestimated by theoretical and experimental works alike. We also run an additional descriptive survey testing standard examples from the linguistic literature and found that at least for some of the examples endorsement appears to be lower than assumed. We end with exploring possible explanations for our results.

As we explore and experience the world, we encounter a wealth of information that needs to be summarized into beliefs for ease of use and recall. Generic beliefs are one fundamental type of beliefs formed in this way: they aggregate observations about the features kinds (or groups) generally have. The role of those beliefs, or generalizations, is to guide us efficiently in our interactions with the world and to allow us to communicate our knowledge to others. Consider for instance the case of mushroom foraging: mushrooms can have a high nutritional and culinary value but it is crucial to avoid the poisonous species. Foragers have for instance formed the belief that “Small-sized *Lepiota* are toxic” because all *Lepiota* that grow to be large are safe to eat while those that are toxic remain small. Notably, this generic belief means that foragers will also pass on the small edible *Lepiota* that haven't finished growing yet. In this way, generic beliefs walk a fine line: if the generalization is too strong, then we incur obvious risks (e.g., deciding that *Lepiota* of all sizes are edible) or lose opportunities (e.g., deciding that mushrooms are toxic); however, when the status of every member of a group is too difficult or too costly to establish (e.g., testing the toxicity of every *Lepiota* we encounter), they can provide a simplified decision-rule that remains tolerant to, and conscious of, the existence of exceptions.

This flexibility of generics with respect to exceptions makes for their usefulness but also for the difficulty in accounting for how they are formed and for what they mean. This paper focuses on generic beliefs in so far as they are expressed by bare plural (or BP) generic sentences like “Birds fly” and “Tigers are striped” (which we take to have the form “*G*s are *f*”, with *G* representing a group and *f* the property ascribed to the group). It examines, both theoretically and empirically, some possible determinants of the formation of generic beliefs and the endorsement of BP generics. In particular, we will investigate whether the endorsement of generic sentences depends on whether the ascribed property *f* is viewed as dangerous or fear-enticing. We will refer to this factor as the *impact* of property *f*.

The paper is structured as follows. Section 1 provides a brief overview of how the philosophical, linguistic and psychological literature have sought to explain the meaning of generics. It then summarizes a proposal made in van Rooij and Schulz (2020) to define the assertability of generics as depending both on frequency information and on considerations about the impact value of the ascribed feature. The Sections 2–5 report on four experimental studies designed to test this theory (one of which is a replication of Cimpian et al., 2010a), in particular the predicted effect of impact on the assertability of generics. We end in Section 6 with a general discussion of our findings and what they could mean for the research on generics.

## 1 How to account for the meaning of generics?

We believe that birds fly, and accept the generic sentence “Birds fly,” even though not all birds do or can fly. How to account for that? A very popular account of generic sentences in linguistics proposes to assume that generic sentences are universal sentences with a restricted domain of quantification: for the generic to be true all the *relevant* or *normal* members of the group *G* of birds, or all the members under *normal* circumstances, need to have the feature *f* of being able to fly (cf. Asher and Morreau, 1995). Unfortunately, this proposal lacks an independent and satisfying account of what relevance and normalcy is, and is therefore unable to truly resolve the problem.

A second prominent approach to generics in the linguistic literature gives up on the idea that all birds need to fly in order for the generic to be true, or acceptable, in a more straightforward way. According to a natural approach (Cohen, 1999, 2004a,b) a generic is true when the **frequency** of members of a group *G* having feature *f* is high.^1^ This raises the following question: what could the threshold for “high” be? Cohen proposes that it should be $\frac{1}{2}$. Thus, according to this theory the generic “birds fly” is true because more than half of all birds can fly. But this proposal to adopting such a fixed threshold immediately gives rise to difficulties when it comes to accounting for the different degrees with which generics allow for exceptions. While it seems that almost all birds have to (be able to) fly for “Birds fly” to be acceptable, there are cases where we are willing to accept generic sentences even when relatively few group members carry the feature in question. For instance, a generic statement like 1 is commonly accepted to be true, even though only 1% of mosquitoes are actually carriers of the virus (Cox, 2004).

(1) Mosquitoes carry the West Nile virus.

In other words, there appear to be other variables that matter for the endorsement of generics besides absolute frequency. One variable that has been proposed in this context is *distinctiveness*: 1 is acceptable because mosquitoes carry the West Nile virus *more often* than any other (relevant) animal. Formally, this means that the generic “Gs are f” can be evaluated as true, or acceptable, in the cases where the probability that some group *G* has feature *f* is greater than the probability that some object in the alternatives to *G* has feature *f* (cf. Cohen, 1999; van Rooij and Schulz, 2020), i.e., *P*(*f*|*G*)>*P*(*f*|*Alt*(*G*)) where *Alt*(*G*) is some contextually salient set of alternatives to *G*. Cohen (1999) includes this idea in his threshold theory by proposing that the threshold for “high” is contextually determined: sometimes it is simply $\frac{1}{2}$, while other times it is *P*(*f*|*Alt*(*G*)) instead.

A third type of proposal for the meaning of generics can be found in the psychological literature (Gelman, 1975; Carlson, 1977; Carlson and Pelletier, 1995; Prasada, 2000; Leslie, 2008a). According to this proposal, generics are not solely based on frequency information. They report on our understanding of the world and consequently are influenced by the ways we link and organize our conceptual knowledge. Because of this our judgments on generics are sensitive to a range of “content-based” factors. At the heart of this type of proposals is the claim that the interpretation of a generic is in part determined by how *striking* a feature is. Here, being striking is taken to subsume a number of factors that appear as relevant for the acceptability of generics. One such factor is that *f* needs to be a (salient) **distinctive** feature for it to be characteristic of the group, or kind, *G*. Note however that this factor can also be captured by the idea that *P*(*f*|*G*)>*P*(*f*|*Alt*(*G*)) discussed above. Another content-based factor that has been proposed is the **dangerousity** of the feature (Leslie, 2008a,b), with more dangerous features leading to increases in the acceptance rate of a generic sentence. Based on this proposal, the acceptability of a generic sentence like 1 can be explained pointing out that carrying malaria is a very striking feature of mosquitoes.

In previous work (van Rooij and Schulz, 2020), we have also argued that the truth or acceptance of generic sentences can be influenced by all three of the factors discussed above: **frequency**, **distinctiveness**, and **dangerousity**. But while in predictions similar, the explanation we offer for why these features matter for the meaning of generic sentences differs from the approach described above. In van Rooij and Schulz (2020) we argue that generic sentence express associations between groups and features we have learned from observing our environment. Thereby, conditions that affect associative learning also matter for the meaning of generic sentences. And indeed, distinctiveness and dangerousity have been shown to influence associative learning. We provide a formal theory, building on approaches from the literature on associative learning, that predicts the assertability of generic sentences from frequency information and *impact*, where a feature is said to have (negative) impact if it is perceived as threat by humans, that is if it comes with a substantially negative utility value.^2^ In Kochari et al. (2020) this theory is put to test, focusing in particular on the interaction of valence and distinctiveness. The present article extents this work by adding an empirical examination of the additional effect of dangerousity, or *(negative) impact*, on the assertability of generic sentence.

Our first goal is to test *whether* impact plays a role for the interpretation of generics. Impact has already been shown to affect how fast humans and animals learn to associate a group with a certain feature by work in the psychology of learning.^3^ Therefore, investigating whether impact matters for the meaning of generics would further bolster future examination of the hypothesis that the meaning of generics is linked to how we learn to associate groups with certain features. Assuming this first objective is met, our second goal for this project will be to study more precisely *how large* the effect of impact on the interpretation of generics is and how it interacts with the other two factors: prevalence and distinctiveness. More specifically, we want to test whether the prediction of Leslie (2008a) and van Rooij and Schulz (2020) bears out that impact together with distinctiveness can account for the assertability/endorsement of generics with low prevalence. Building on the formal theory mentioned above, we derive the following empirical hypotheses:

H1: The assertability of a generic statement will be positively affected by the prevalence of the feature in the group described by the statement (or target prevalence). More precisely, increases in target prevalence will lead to a higher probability of the statement being perceived as assertable, or being endorsed.
H2: The assertability of a generic statement will be negatively affected by the prevalence of the feature in the group that is presented as alternative to the described group (or alternative prevalence). For a fixed target prevalence, increasing the alternative prevalence will decrease the distinctiveness of the generic and lead to a lower probability of the statement being perceived as assertable, or being endorsed.
H3: The assertability of a generic statement will be positively affected by the impact value of the feature being ascribed. A feature with a high impact value, in particular a feature presented as highly dangerous, will lead to a higher probability of the statement being perceived as assertable, or being endorsed.

This is not the first paper studying the effect of impact on the truth/acceptability of generic sentences. Next to evidence from the literature on associative learning, there is also some evidence from work on generics that negative impact (or impact for short) does play a role for the meaning of generics. In Cimpian et al. (2010b) the authors provided participants statistical information about the prevalence of a feature in fictional animal kinds and contrasted neutral features with striking (distinctive and high impact) features at varying prevalence levels. Their analysis of participants' truth ratings indicated that striking generics were more likely to be endorsed than neutral generics at low prevalence levels, while at higher prevalence levels endorsement levels did not appear to differ, i.e., they were high for both types of generic statements. However, the reported endorsement rates for striking generics with low prevalence were only weakly positive. More recently, Bian and Cimpian (2021) replicated the finding that endorsement of generics for lower levels of prevalence increases when the feature is striking or dangerous^4^ and extended it to the case of habitual generics. In these studies the effect of distinctiveness and dangerousity was smaller than in Cimpian et al. (2010b) and not sufficient to explain the endorsement of low prevalence generics with striking features.

On the other hand, there are a number of studies that report that generalizations about individuals based on negative behaviors are sometimes less likely (Heyman and Giles, 2004; Lockhart et al., 2002) or don't find an effect of impact on generalization (rholes_childrens_1984).^5^ Most notably, Cella et al. (2022) investigated whether a number of previously observed effects on the acceptability of generics, including negative impact, could be extended to generics about human groups but their reported results did not suggest that the endorsement of negative impact generics was significantly higher, even at lower prevalence levels. They did, however, observe that when prompted with a generic sentence participants would assign a lower probability of the group having the feature for striking than for non-striking features. Lazaridou-Chatzigoga et al. (2019) studied the effect of negative impact features on participants' likelihood to extend a generalization to new members of a kind and found, similarly, that both children and adults were less likely to extend a striking feature (rather than a neutral one) to a new individual. In sum, the empirical evidence for the role of negative impact features on generic endorsement is still rather mixed and limited, with the studies reported in the literature leaving various questions open. We hope that this work will answer at least some of them.

## 2 Experiment 1

Experiment 1 asked participants to rate the assertability of generic statements with varying impact values on the basis of visual information regarding the prevalence of the feature in the target group and in a relevant alternative class. Our goal was 2-fold: (1) testing the hypothesis that probabilistic information plays a role on the formation of generic beliefs, with the prevalence of the feature in the target group having a positive effect on the endorsement of a generic belief and the prevalence of the feature in the alternative group having a negative effect on that same endorsement; (2) investigating the claim that generics regarding features with a high impact value (i.e., dangerous features) are more likely to be endorsed than their lower value counterparts.

**Figure 1:**
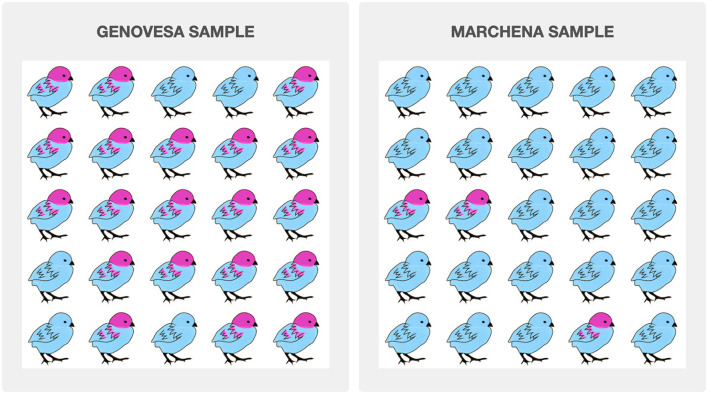
Stimuli example for the low impact condition. This example has a high contrast level: 92% in the Genovesa sample vs. 12% in the Marchena sample.

To achieve this 2-fold goal, we ran an experiment based on the method in Kochari et al. (2020) (who built on Bordalo et al., 2016), but extended it to include items with a low and high impact value as well as a wider range of prevalence levels. The experimental setup uses visual representations of statistical information in the form of samples drawn from a relevant group, where each object in the sample is marked for whether or not it has a relevant feature (see Figure 1). The motivation behind using such pictures is to model an actual experience of observing frequencies instead of providing a textual description, which activates additional higher order cognitive mechanisms necessary to process the verbal description. In the design used here the sample information is presented simultaneously. There exists extensive research on the difference of simultaneous or sequential presentation of information for learning and generalization. In a number of studies it was observed that simultaneous presentation leads to better generalization results than sequential presentation (Son et al., 2011; Vlach et al., 2012; Lawson, 2017). Studies on associative learning tasks have shown that simultaneous presentation provide better support for identifying cues that are shared by exemplars, while sequential presentation support identification of properties or cues that differentiate exemplars (Lipsitt, 1961; Williams and Ackerman, 1971; Rescorla, 1980). In general, it appears that the type of presentation (sequential or simultaneous) leads to different modi of information processing. In case of simultaneous presentation the focus lies on identify underlying similarities between presented items (Gentner and Namy, 1999, 2006; Namy and Gentner, 2002; Boroditsky, 2007), whereas in case of sequential presentation the attention is rather on discrimination between presented items (Lipsitt, 1961; Rescorla, 1980). In our tasks both cognitive tasks play an important role. We decided to use simultaneous presentation, because it allowed us to test participants on a wider set of conditions.

### 2.1 Method

All methods and analyses were preregistered on the Open Science Framework. The preregistration documents for this experiment can be found at https://osf.io/gbvrd/?view_only=b6de6ce94ac94a07952ebe3cd7c3a022. The data, analyses scripts, figures and model fits can be found at https://osf.io/d8h47/?view_only=ef750f8238a7470eaee777cfb360a0e5.

#### 2.1.1 Participants

Participants were recruited through the Prolific.co platform. Three hundred and fifty-nine participants were recruited and after excluding incomplete response sets as well as participants who failed attention checks, we were left with data from 329 participants (207 women, 102 men; M_age_ = 39.7, SD_age_ = 12.8).^6^

#### 2.1.2 Materials and procedure

The study was run online using the Qualtrics platform and adopted a 2 × 8 (Impact × Contrast Pairs) within subject design, where each participant received sixteen trial items.

**Figure 2:**
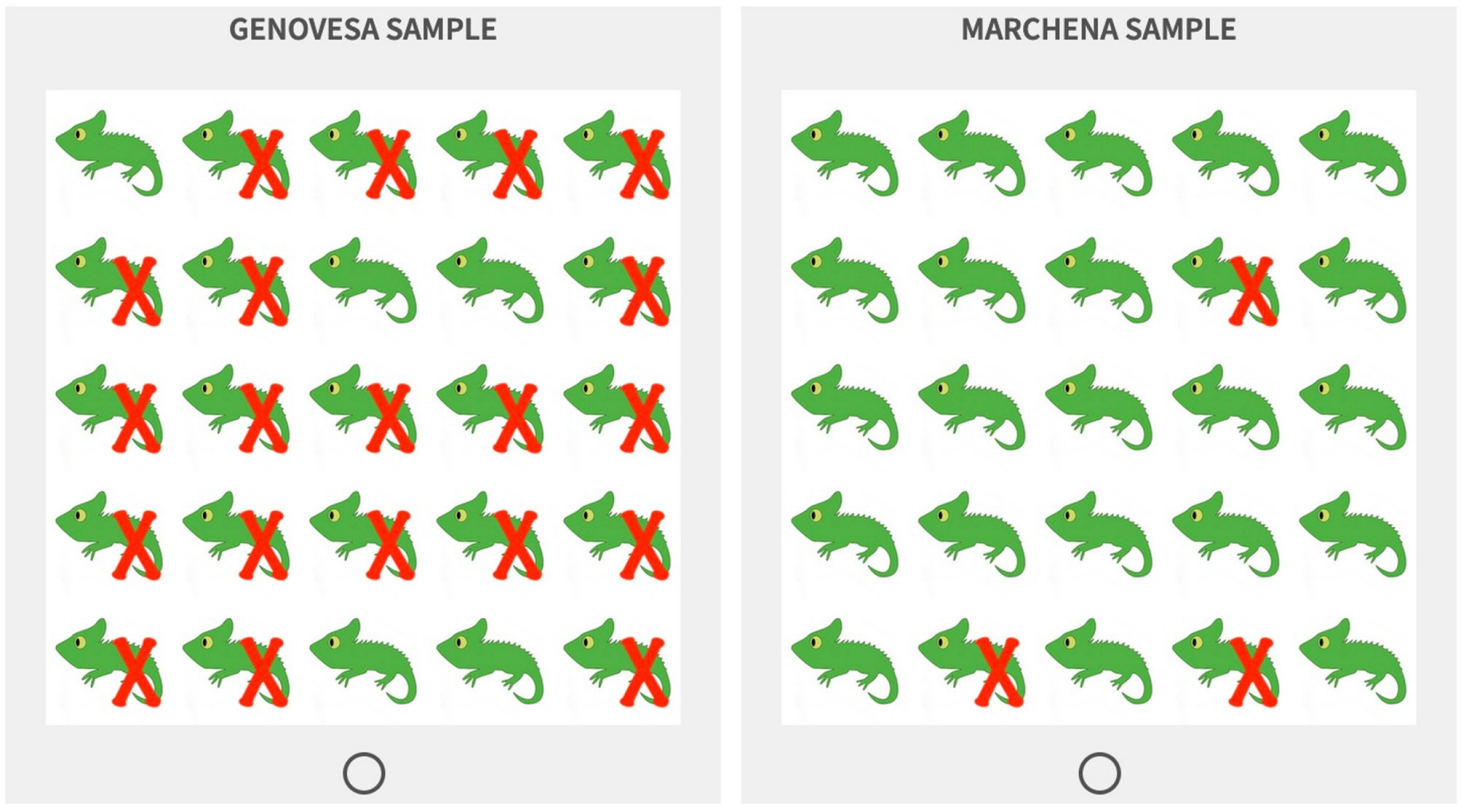
Stimuli example for the high impact condition with the same contrast level as Figure 1: 92% in the Genovesa sample vs. 12% in the Marchena sample.

As explained above, visual stimuli were used to communicate information about the prevalence of the feature in a group. These visual stimuli took the form of 5 × 5 grids, with samples of fictive animal species. Two samples of the same species are placed next to each other to simulate contrast: the comparison of one group relative to a set of alternatives. In the background story provided to the participants it is explained that the samples are taken from two different islands of the Galapagos Islands. Each animal was represented either with or without a specific feature. In the low impact version, a visual feature was added to or modified in the plain picture of the animal (e.g., the red head and red feathers of the bird in Figure 1). In the high impact version, a red cross was superimposed on the picture to indicate the presence of the high impact feature, given that those features corresponded to characteristics that could not be directly represented on the illustrations (e.g., behavioral predispositions, tendencies to carry certain diseases, etc., see Figure 2). Finally, grids were randomly generated for four prevalence levels (0.12, 0.2, 0.8, and 0.92) and were then assembled into pairs with different levels of contrast: six pairs with varying contrast (0.12 vs. 0.8, 0.2 vs. 0.92, 0.12 vs. 0.2, and 0.8 vs. 0.92) and four pairs with no contrast (one for each prevalence level: 0.12 vs. 0.12 and so on). Examples for all eight contrast conditions are provided in the Appendix.

The study was composed of three main parts: after providing informed consent, participants received instructions about the setting of the task and completed three practice trials. Next, they completed the sixteen test trials and then responded to a few debriefing questions.

The first part started with an introduction to the experimental setting. Participants were shown a map of the Galapagos islands that highlighted the Genovesa and the Marchena islands and were told that they would receive information about animal species that live on those islands and would be asked to evaluate judgements about those species. In particular, it was underlined that those two islands were inhabited by the same species of animals, except that genetic differences had lead them to now exhibit discrepancies in appearance or behavior. Participants then completed three practice trials (one in the low impact condition and two in the high impact conditions) using an animal that was not included in the test trials. They were also given instructions about the meaning of the assertability scale and about the meaning of the red cross used in the high impact condition. Finally, participants completed one comprehension check that verified their understanding of what the red cross indicates.

In the second part of the experiment, participants completed sixteen test trials and one attention check trial (which appeared midway through the survey and used an animal that was not included in the test trials). In each test trial, participants were first shown a pair of side-by-side grids (generated as described above) under a legend that indicated the feature of interest and the name of the species (e.g., “Distribution of dark blue wings in bats” or “Distribution of highly aggressive bats” for the low impact and high impact conditions, respectively). Each grid also had a header indicating the origin of the sample, i.e., “Genovesa sample” and “Marchena sample,” with the Genovesa sample always appearing on the left. Participants could observe the stimuli for a maximum of 10 s before being automatically brought over to the next page, where they were asked to rate whether two generics statements describing the samples [e.g., “Genovesa (resp. Marchena) bats have dark blue wings.”] were assertable, with the statement about the Genovesa sample always appearing first. Participants were prompted with “Are the sentences below assertable?” and responses were collected on a 7-point Likert scale ranging from “Highly unassertable” to “Highly assertable” with “Neither assertable nor unassertable” as a middle point. Participants received half of the test items in the low impact condition and half of the items in the high impact condition, and for each condition, they received eight different contrast pairs, as described above. The order of the test items, the order of the grids for each test item (which grid was described as the Genovesa or the Marchena sample, respectively) as well as the animal used in each test item were all fully randomized across participants.

In the final part of the experiment, participants were asked for feedback, completed a final attention check (they were asked whether they responded seriously to the questions in the study) and finally received a debriefing sheet.

### 2.2 Results

#### 2.2.1 Analytic approach

To test our predictions regarding the effect of statistical information and impact value on assertability judgments for a generic statement, we fit a Bayesian regression model with the R package brms (Bürkner, 2017) and the probabilistic language Stan (Carpenter et al., 2017), which uses Markov Chain Monte Carlo algorithms. A Bayesian analysis estimates model parameters (in this case, the cumulative probability of each degree of assertability for a generic in a given impact condition and for a given target and alternative prevalence level) as probability distributions, with the joint probability distribution of the data, *y*, and a given parameter, θ, being computed via the prior probability of *θ* and the probability *p*(*y*|*θ*):


p(y,θ)   =   p(y|θ)×p(θ).

This result is derived from Bayes' Rule, which serves to calculate the posterior probability, *p*(*θ*|*y*):


p(θ|y) ∝ p(y|θ)×p(θ) = p(y,θ).

This posterior probability distribution can be interpreted as indicating the relative plausibility of possible values of the parameter *θ*, conditional on the prior probability of that parameter, the probability distribution of the responses (or likelihood function), and the data itself.

Given that responses were provided on a 7-point Likert scale, we opted to analyze the data using an ordinal logistic regression model. An ordinal regression model assumes responses to have resulted from the categorization of a latent continuous variable (e.g., the assertability of a generic statement) which is divided by respondents into bins (corresponding, for instance, to each point on a Likert scale) of possibly varying sizes (Bürkner and Vuorre, 2019). Importantly, the distance between two points on a Likert scale cannot be assumed to be the same for all pairs of contiguous points on the scale nor can it be assumed to be the same for all participants, two features which are incompatible with the assumptions of linear regression and which ordinal regression is specifically meant to account for (see e.g., Liddell and Kruschke, 2018; Bürkner and Vuorre, 2019).

The models reported in Experiment 1 (and Experiments 2 and 3 below) estimate how the explanatory variables influence the logit-transformed probability of respondents selecting a given response point on the response scale. The logit-transformation converts a probability *p* (which is, by definition, restricted to the 0–1 range) into a log odds ratio by taking the logarithm of the ratio between *p* and 1−*p*. A log odds ratio of 0 means that *p* and 1−*p* are equal, a positive log odds ratio means that *p* is larger than 1−*p*, and a negative log odds ratio means that *p* is smaller than 1−*p*.

We also specified weakly informative priors to indicate extremely high or extremely low probability estimates for any given response as unlikely and to indicate that extreme effects for the main predictors are unlikely while remaining agnostic to the direction of these effects. Finally, because we used a repeated measures design where participants provided ratings for multiple items and where items were rated by multiple participants, we also included a (hierarchical) mixed-effects structure to our model, which estimates how group-level (or random) effects deviate from population-level (or main) effects and accounts for possible correlations in responses provided by the same participant or to the same item.

#### 2.2.2 Impact and prevalence model

Our model for Experiment 1 regressed assertability judgments for a generic statement on the prevalence of the feature in the target group and in the alternative group (abbreviated to “Target” and “Alternative” respectively), which were defined as numeric variables and centered on 50%; and on impact condition (viz. “Impact”), which was defined as a categorical variable with “Low impact” as the reference level. The model further included an interaction term between each of the prevalence terms and the Impact term:


**Model 1**: Assertability ~ (Target +
 Alternative) * Impact +
 ((Target + Alternative) * Impact |
 Participant) +
 ((Target + Alternative) * Impact | Item)


MCMC diagnostics indicated sufficient mixing of the chains, sufficiently high bulk and tail effective sample size values, and an $\hat{R}$ convergence diagnostic of 1.01 or below for all parameters, which is below the maximum recommended value of 1.05 (Vehtari et al., 2021b).

**Figure 3:**
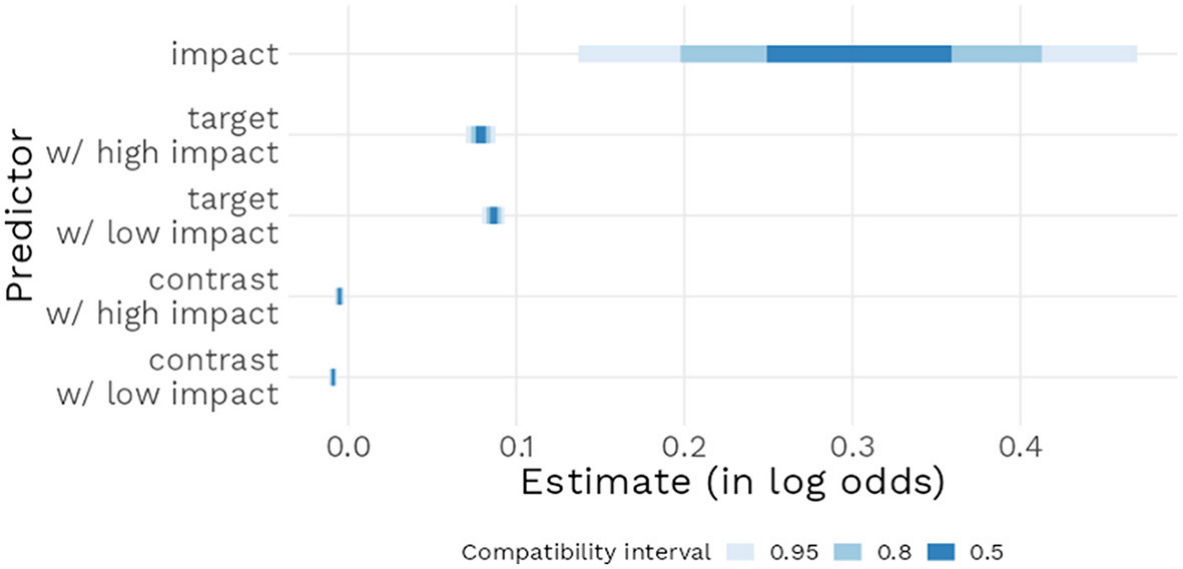
Posterior distribution of the relative effect of main model parameters, with 95% compatibility interval.

Model 1 estimated a positive effect of High impact compared to Low impact [*b* = 0.30, 95% CI (0.13 : 0.46)] in the case where target and alternative prevalence were at 50%. This model also estimated that an increase in Target prevalence had a positive effect [*b* = 0.086, 95% CI (0.079 : 0.093)] in the Low impact condition; when in the High impact condition, with the addition of the corresponding interaction estimate, the effect of target prevalence [*b* = 0.079, 95% CI (0.07 : 0.087)] was largely similar (see Figure 3). These results, after application of the inverse logit transformation, mean that a 1% increase in the target prevalence increased the probability of the generic being perceived as assertable, and that this effect did not increase further when the impact value of the feature was increased to high. Finally, the model estimated that increasing alternative prevalence (such an increase leads either to a smaller difference between target and alternative groups or to the prevalence of the alternative group becoming higher than the prevalence of the target group) had a small negative effect in the Low impact condition [*b* = −0.009, 95% CI (−0.011 : −0.006)]; when in the High impact condition, with the addition of the corresponding interaction estimate, alternative prevalence had a negative effect of similar size [*b* = −0.005, 95% CI (−0.008 : −40.002)]. To guide the interpretation of the Target and Alternative estimates, note that their units were taken to be the percentage: their respective estimates may therefore seem small at the 1% scale but their effects will naturally become larger if one considers larger (and certainly more meaningful) prevalence increases, for instance of 10 or 20%.

**Figure 4:**
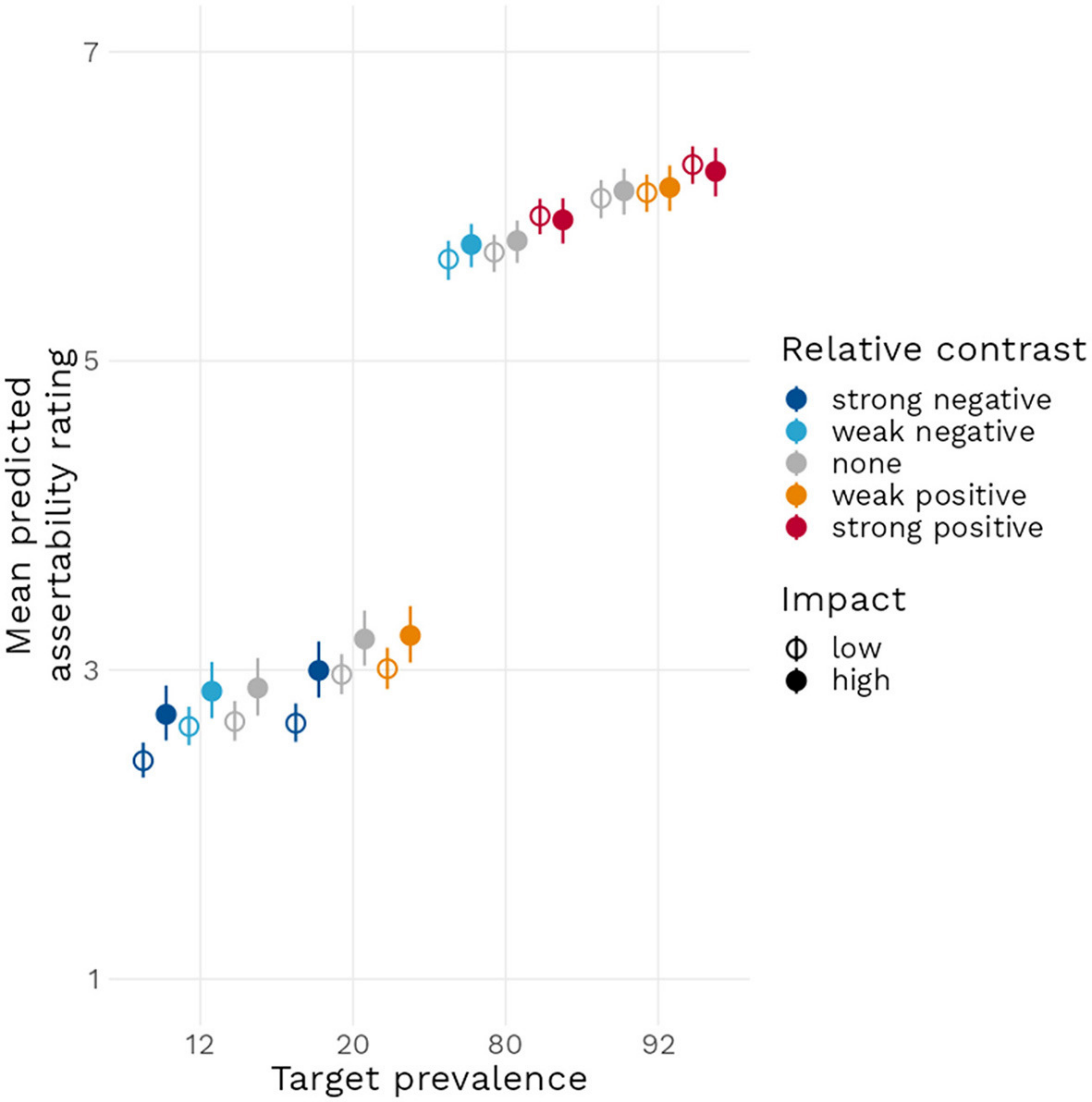
Conditionals effect plot with 95% compatibility interval for the effect of increasing target prevalence, both in terms of impact and of relative contrast, on the mean predicted assertability rating.

Because of the complexity of the data and model structure and for ease of representation, we chose to reorganize the two prevalence conditions in terms of relative contrast (i.e., distinctiveness) as follows: strong negative (12 vs. 80 and 20 vs. 92%) and weak negative (12 vs. 20 and 80 vs. 92%) relative contrast, neutral relative contrast (12 vs. 12% and so on), weak positive (20 vs. 12 and 92 vs. 80%), and strong positive (80 vs. 12 and 92 vs. 20%) relative contrast. We then plotted the model predicted conditional effects of relative contrast and impact on the mean predicted assertability rating of a generic. Figure 4 reveals that mean predicted assertability ratings increase as a function of target prevalence and of relative contrast and that a higher impact value tends to increase mean predicted assertability, although this effect is stronger when target prevalence is inferior to 50%.

Finally, we cross-validated Model 1 using the loo package which performs leave-one-out cross-validation (Vehtari et al., 2021a). This method provides estimates of the point-wise out-of-sample prediction accuracy (or ELPD) of a model, as well as approximations of the standard error for the estimated prediction error of a model, thereby enabling comparisons in predictive accuracy between models. We cross-validated Model 1 by comparing it with five restricted models (see Table 1): a model that included all three predictors but no interaction term between them (Model TCI), a model that only included target and alternative prevalence (Model TC), a model that only included target prevalence, impact and their interaction (Model TI*), a model that only included target prevalence and impact (Model TI) and finally a model that only included target prevalence (Model T). The difference in ELPD between Model 1 and any of the other models was seven to twenty times larger than the standard error of that difference, indicating that Model 1 had a better predictive performance than a model that would not attribute a role to impact nor to target nor to alternative prevalence.

**Table 1:** Cross-validation comparisons of the models.

**Model**	**Δ_ELPD_**	**(SE)**
1	**0.0**	**0.0**
TI^*^	−151.9	20.2
TCI	−1,312.6	69.6
TI	−1,372.4	69.7
TC	−1,383.7	70.6
T	−1,440.3	70.7

For each cross-validation comparison, the Δ_ELPD_ is the difference in ELPD between each model and the model with the largest ELPD (indicated in bold face). The Δ_ELPD_ for the best model is always 0 given that it is the difference between that model and itself. SE corresponds to the standard error of the difference in ELPD between two models and indicates the amount of uncertainty present in the comparison. The Δ_ELPD_ must be several times larger than the SE for the comparison to indicate meaningful differences in predictive performance between two models.

### 2.3 Discussion

Following the expectations of the large majority of theories regarding generics, including ours (H1), this experiment first found that increasing the target prevalence of a feature lead to generics becoming more likely to receive higher assertability ratings. It also revealed supporting evidence in favor of an effect of relative contrast (H2): when the difference between the prevalence in the target group and alternative group decreased or even became negative, generics became more likely to receive lower assertability ratings, both in the Low and High Impact conditions.

Finally, and according to our predictions (H3), impact value increased the likelihood of a generic receiving higher assertability ratings. This result, however, requires mitigation as well: the role of impact and distinctiveness in our theory (as well as in those theories presented in Cimpian et al., 2010b) is to account for the common endorsement of high value generics that have a low prevalence, such as “Mosquitoes carry malaria”. Examining Figure 4 reveals that at low target prevalence, the mean predicted rating increases when impact is high, but it does not increase so much that it would become likely to receive a positive assertability rating (i.e., a rating above “4” on the assertability scale). Adding relative contrast (weak positive contrast in this case) increases asserability again, but still not to a level of positive assertability. This observation is confirmed by examining the size of the estimates: the impact estimate is approximately three to four times larger than the target prevalence estimate, meaning that going from a neutral feature to a high impact one only would have as much predicted effect as a relatively small increase in prevalence of 3–4%. Comparing with similar studies in the literature we find that the effects of impact and distinctiveness we observed are smaller than those of Cimpian et al., 2010a. Though, as already noticed in the introduction, also in this study low prevalence examples with high strikingness barely reach an on average positive truth value. As was also pointed out in the introductions, Bian and Cimpian, 2021 found, using the same experimental design as Cimpian et al., 2010a, substantially smaller effects of impact and distinctiveness. In fact, their results appear very similar to our findings in this experiment.^7^ Also in Bian and Cimpian, 2021 low prevalence generics with high distinctiveness or high impact are on average not considered true by the participants of the study.^8^

In light of these results, we chose to simplify our next experiment by not providing participants with information about alternative groups anymore and instead to focus on the role of impact on assertability ratings. We sought to examine whether the small effect of impact revealed by Model 1 might have been caused by issues in our experimental manipulation. One possible weakness of our design could have been that given the way we marked high impact features, they were not distinguishable as such in the visual material. These features were all marked as crosses on the objects in the samples. But to process what the cross stands for the participants had to read the descriptions given with the pictures. It might be that after a couple of trials the participants reverted to answer the question just by looking at the frequency information in the pictures without reading the descriptions. In this case they would not have taken into account the difference between low impact and high impact condition anymore. To exclude this possibility, we chose to replace the visual stimuli by textual stimuli. We also decided to add to each generic details that would reinforce the dangerousity of the feature and to collect participants' subjective dangerousity ratings for each generic, which would verify whether participants were appropriately sensitive to the impact manipulation. In doing so, our experimental design also became more similar to the one used Cimpian et al. (2010a), which would allow a more direct comparison.

## 3 Experiment 2

In Experiment 2, participants were asked to rate the assertability of generic statements and, in a later task, to evaluate the dangerosity of the features being generalized. Those generic statements varied by their impact value and by the prevalence of the feature in the group. Participants were provided information regarding the feature's prevalence and characteristics via textual information. Our goal was again to determine the role of statistical information about the prevalence of the feature on the assertability of a generic and to examine how generalized features with a high impact value affected that same assertability. Furthermore, by collecting dangerosity ratings, we sought to confirm that participants were sensitive to the impact value manipulation and we also hoped to achieve a more fine-grained understanding of how value considerations affect generic endorsement.

### 3.1 Method

Preregistration documents for this experiment can be found at https://osf.io/c7fbr/?view_only=7543af24eaa441a89c8c3dcf107f8a88.

#### 3.1.1 Participants

Three hundred and fifty-nine participants were recruited through the Prolific.co platform and after excluding incomplete response sets as well as participants who failed attention checks, we were left with data from 289 participants (195 women, 78 men; M_age_ = 38.66, SD_age_ = 13.9).^9^

#### 3.1.2 Materials and procedure

The study was run online using the Qualtrics platform and adopted a 2 × 5 (Impact × Prevalence) within subject design, where each participant received ten trial items.

Borrowing from the design from Cimpian et al. (2010a), the visual stimuli used in Experiment 1 were replaced by textual ones: a generic statement ascribing a feature to an animal species and an accompanying statement that provided further detail about the feature in question. Half of the items were presented in a high impact condition, where the feature ascribed was expected to elicit fear or disgust, with the accompanying statement emphasizing or explaining the dangerosity of the feature. The rest of the items were presented in a low impact condition, where the feature ascribed had a neutral valence and the accompanying statement described neutral characteristics of the animal under consideration.

The experiment was composed of four parts: an assertability questionnaire, a distractor task, a dangerosity questionnaire and a debriefing questionnaire, which were presented in that order. At the beginning of the experiment, participants provided informed consent and were briefly introduced to the setting of the experiment.

Next, the assertability questionnaire began with instructions about what it means for a statement to be assertable, a training example and a comprehension check. Participants then received ten items (five in the high impact condition and five in the low impact condition) for five different prevalence levels: 0, 12, 32, 68, 92%, which were presented in a randomized order. For each item, participants were given the prevalence of the feature among the group (e.g., “On the Galapagos islands, 32% of stag beetles have a venom-filled sting.”) with an accompanying statement that provided additional information about the feature in question (e.g., “This sting causes your skin to turn black and die from necrosis.”). Participants were then asked to rate the assertability of the corresponding generic statement (“Galapagos stag beetles have a venom-filled sting.”) on a 7-point Likert scale ranging from the “Highly unassertable” to “Highly assertable”. Participants also received an attention check midway through this part in which they were asked to rate the assertability of a generic statement where the prevalence of the feature was 100%.

Before moving on to the dangerosity questionnaire, participants completed a short distractor task in which they were asked to find a list of words in a grid of letters (word search task). Participants then received the dangerosity questionnaire, which began with brief instructions about what it means for an animal to be dangerous and with a practice example. Participants were then shown the same ten items from the assertability questionnaire but *without* the prevalence information and were asked to rate the dangerosity of each animal on a 7-point Likert scale ranging from “Entirely harmless” to “Extremely dangerous”. Midway through this part, participants received a second attention check.

Finally, participants were asked for feedback, completed a final attention check (they were asked whether they responded seriously to the questions in the study) and received a debriefing sheet.

### 3.2 Results

**Figure 5:**
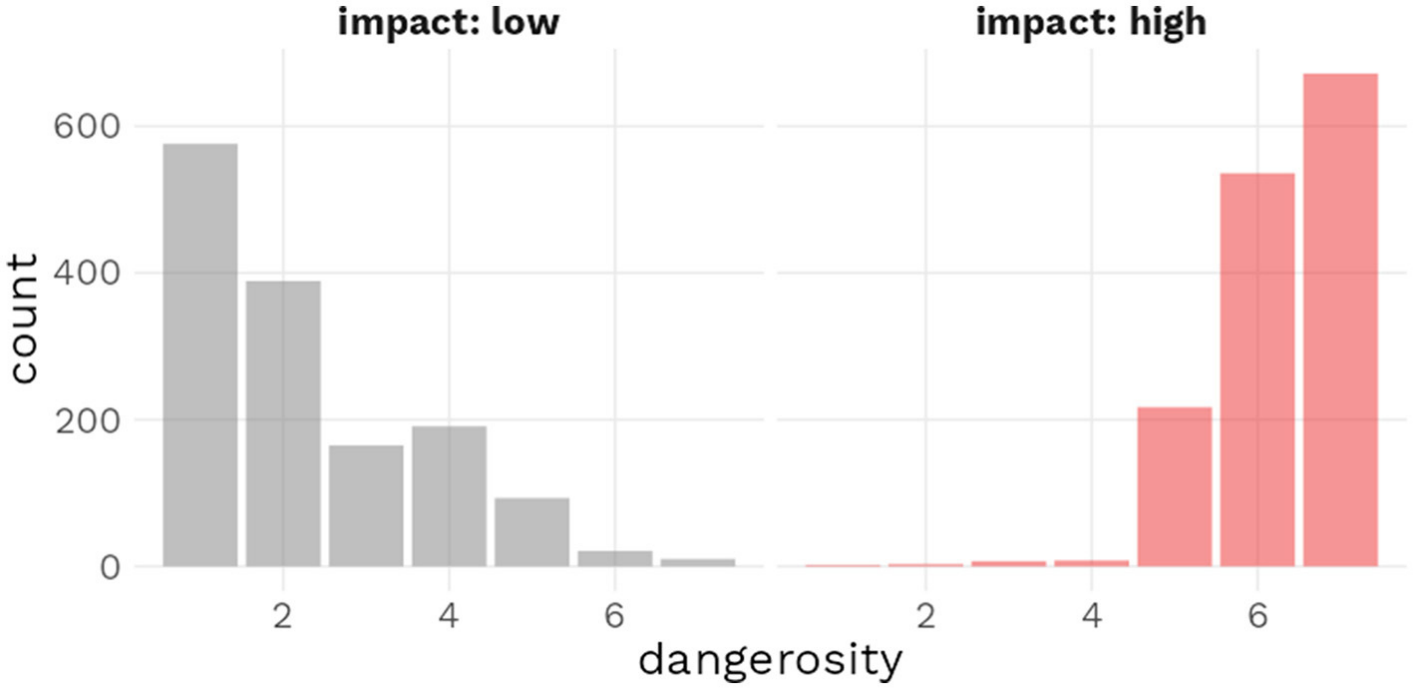
Histogram of dangerosity ratings depending on experimenter-defined impact condition.

We confirmed the success of the impact value manipulation with Figure 5. The histogram reveals that the large majority of dangerosity ratings for items in the low impact condition were concentrated on the lower half of the dangerosity scale; meanwhile, items in the high impact condition received almost exclusively received dangerosity ratings on the upper half of the dangerosity scale.

Given that responses were again provided on a Likert scale, we fit a Bayesian ordinal logistic regression model that regressed assertability judgments for a generic statement on the prevalence of the feature in the group (centered on 50%), the dangerosity of the feature according to participants and the interaction of those two terms. Dangerosity was specified as a monotonic predictor, given that responses to the dangerosity task were collected on an ordinal Likert scale. Monotonic effects are the recommended approach for modeling predictors where one can expect a monotonic (increasing or decreasing) relationship between the predictor and the response but where intervals on the scale cannot be assumed to be equidistant and should therefore be allowed to have varying effects on the response (Bürkner and Charpentier, 2020). The model again included a mixed-effects structure and was defined as follows:


**Model 2**: Assertability ~ Prevalence *
 mo(Dangerosity) +
 (Prevalence * mo(Dangerosity) |
 Participant) +
 (Prevalence * mo(Dangerosity) | Item)


MCMC diagnostics indicated sufficient mixing of the chains, sufficiently high bulk and tail effective sample size values, and an $\hat{R}$ convergence diagnostic of 1.0 or below for all parameters.

**Figure 6:**
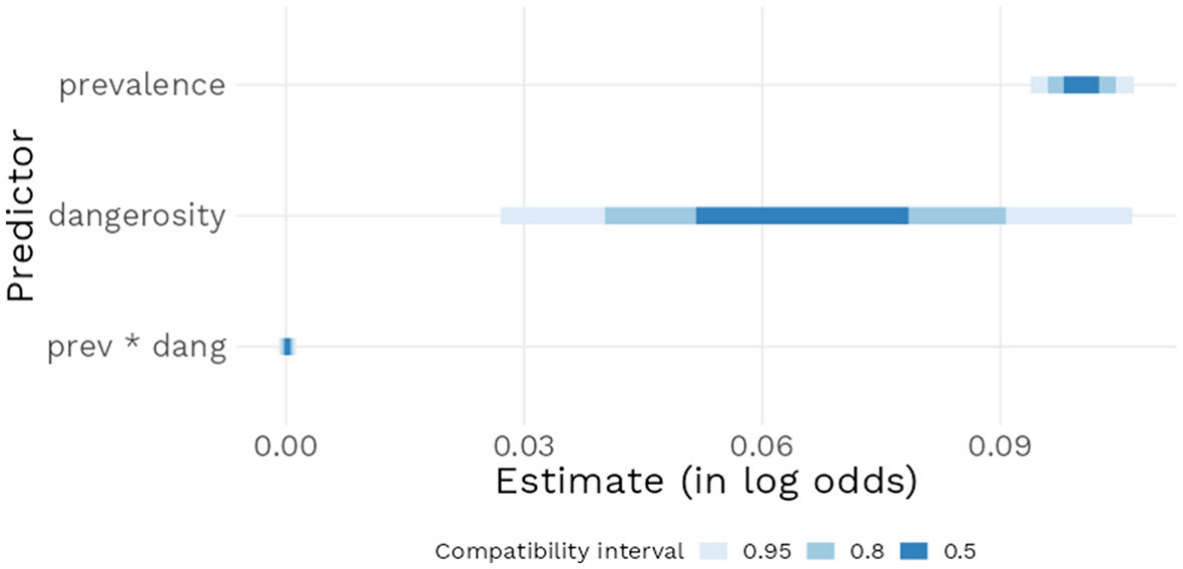
Posterior distributions of main model parameters with 95% compatbility interval.

**Figure 7:**
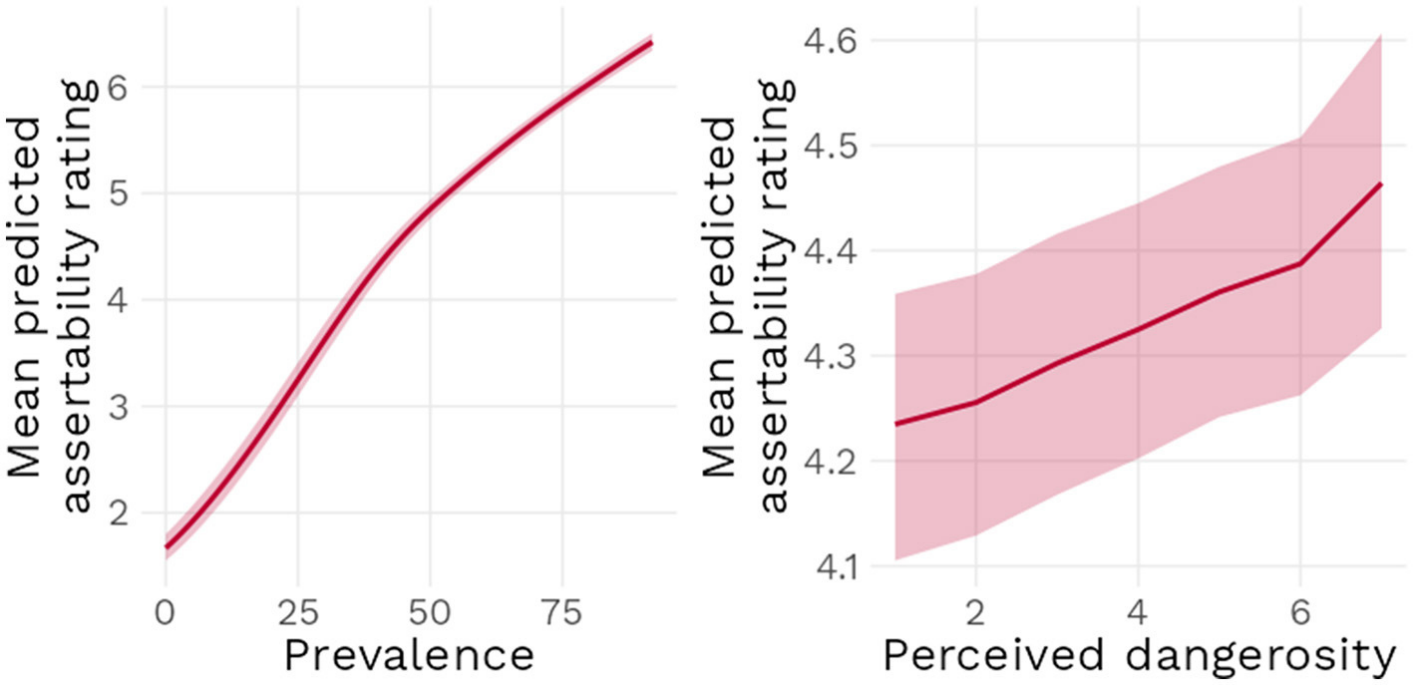
Mean predicted assertability rating as a function of prevalence when dangerosity is rated at its lowest point **(Left)** and perceived dangerosity when prevalence is at 50% **(Right)**, with 95% compatibility interval.

Model 2 found a positive effect of increasing dangerosity [*b* = 0.07, 95% CI (0.03 : 0.11)] when prevalence was at 50%, as well as a positive effect of increasing prevalence [*b* = 0.10, 95% CI (0.09 : 0.11)] when Dangerosity was at its lowest. The interaction term was estimated to have a null effect [*b* = 0.0, 95% CI (0.0 : 0.0)], indicating that increasing both Dangerosity and prevalence at the same time did not have any further *additional* effect on assertability ratings (see Figure 6). Figure 7 underlines these results: as prevalence increases, mean predicted assertability ratings increase as well. Higher dangerosity ratings are also associated with higher predicted mean ratings, but note that this effect was relatively limited by the range of the scale: at 0% prevalence, the maximum possible increase in dangerosity of seven points at best lead to a mean predicted increase in assertability rating of half a point.

We cross-validated Model 2 by comparing it with four restricted models (see Table 2): a model that included both terms but not their interaction (Model DP); a model that included only one of the two predictor terms (Model D and Model P); a null model that included no predictors (Model 0). Model DP was revealed to have a better predictive power than Model 2 (the standard deviation was over eight times larger than the ELDP difference), indicating that the addition of the interaction term increased model complexity without contributing meaningfully to predictive power, and to also have a better predictive power than Model D (the standard deviation was over twenty-five times larger than the ELDP difference), suggesting that the inclusion of prevalence in the model meaningfully improved its predictive power.

**Table 2:** Cross-validation comparisons of the models.

**Model**	**Δ_ELPD_**	**(SE)**	**Model**	**Δ_ELPD_**	**(SE)**
DP	**0.0**	**0.0**	IP	**0.0**	**0.0**
2	−5.8	0.7	2a	−3.0	0.9
P	−8.1	3.9	P	−6.4	3.9
D	−1,935.7	52.3	0	1,935.0	52.5
0	−1,336.8	52.3	I	1,935.9	52.4

For each cross-validation comparison, the Δ_ELPD_ is the difference in ELPD between each model and the model with the largest ELPD (indicated in bold face). The Δ_ELPD_ for the best model is always 0 given that it is the difference between that model and itself. SE corresponds to the standard error of the difference in ELPD between two models and indicates the amount of uncertainty present in the comparison. The Δ_ELPD_ must be several times larger than the SE for the comparison to indicate meaningful differences in predictive performance between two models.

For ease of comparison with Model 1, and Model 3 below, we also fit Model 2A, which replaced the Dangerosity term by an Impact term corresponding to the impact manipulation, defined as follows:


**Model 2A**: Assertability ~ Prevalence * Impact
 + (Prevalence * Impact | Participant) +
 (Prevalence * Impact | Item)


MCMC diagnostics were again appropriate for all parameters. This model found an identical effect of prevalence on assertability ratings and a larger positive effect of Impact [*b* = 0.29, 95% CI (0.11 : 0.47)], which can be explained by the fact that Impact was a binary variable whereas Dangerosity was a 7-point scale. The interaction term was again estimated to have a null effect. Model 2A received the same cross-validation procedure as Model 2, with the Impact term (I) replacing the Dangerosity term in each model. Results were sensibly similar to those for Model 2.

### 3.3 Discussion

The findings revealed by Model 2 provide further support for our two hypotheses, H1 and H3: higher assertability ratings became more likely when prevalence increased and higher ratings of perceived dangerosity were also correlated with higher assertability ratings. Furthermore, the estimates from Model 2A underline how well Experiment 2 replicated the results of Experiment 1 regarding H1 and H3: not only were the coefficients for prevalence and impact value in the same direction, they also had sensibly similar sizes. These results indicate that participants were generally sensitive to the differences in dangerosity between the low impact and high impact conditions and suggest that conveying prevalence through numerical percentages or through static visual arrays of pictograms leads to a similar effect of prevalence on assertability ratings.

Having confirmed that the small effect of impact on assertability was not due to our experimental manipulation being too weak, we turned to another possibility for why our results deviate from those of Cimpian et al., 2010a: we had chosen to collect assertability ratings as a response variable because this concept reflected more accurately the semantic of generics, which are not properly true or false but rather more or less appropriate to state, or *assert*, given the context. However, arguably, participants were unlikely to be familiar with the notion of “being assertable” and this might have affected their responses. Moreover, the training on the meaning of “being assertable” that was included in Experiment 2 used the example of how “This man is bald.” becomes less and less assertable as the number of hairs on a bald man's head increases to explain the meaning of the construct. Because this example directly connected a numerical value with the assertability of a statement, we might have primed participants to focus on numerical information when evaluating the assertability of a generic, to the detriment of more qualitative aspects, such as how dangerous the generic feature was.^10^ In light of this issue, we chose to replace our assertability task by a much more familiar truth task in our next experiment.

A secondary motivation were the results from Study 3a in Cella et al. (2022): the authors replicated the design from Cimpian et al. (2010a) with a “Truth-Conditions” task and sought to extend the original's results by varying not only the prevalence and the “property valence” (their terminology for our notion of impact) but also the domain (animal or social) of the generic. The estimated effect of Property valence according to their analyses did not achieve statistical significance. From this perspective, we might then expect to find that our observed effect of impact on assertability, instead of being smaller than expected, would simply not appear when judgments were collected on a True/False scale instead.

## 4 Experiment 3

Experiment 3 was a close replication of Experiment 2 with two important exceptions^11^: first, participants were asked to evaluate the truth, rather than the assertability, of the generic statements they received, and provided answers on a True/False response scale; second, the dangerosity questionnaire was not included because Experiment 2 had already confirmed that participants were appropriately sensitive to the impact manipulation. Like Experiment 2, this experiment had a 2 × 5 (Impact × Target Prevalence) within subject design, with each participant receiving ten trial items.

### 4.1 Participants

Three hundred and fifty-six participants were recruited again through the Prolific.co platform and after excluding incomplete response sets as well as participants who failed attention checks, we were left with data from 321 participants (188 women, 120 men; M_age_ = 44.05, SD_age_ = 13.5) (see text footnote ^4^).

### 4.2 Results

Given that responses to this experiment were collected on a binary response scale, we decided to analyze the data using a Bernoulli regression model, which estimated the logit-transformed probability of a generic being endorsed given the Impact condition and prevalence it was shown in. Impact was again specified as a categorical variable, with “Low Impact” as the reference level, the Prevalence term was centered on 50 and a mixed-effects structure was included. Model 3 had the following definition^12^:


**Model 3**: Endorsement ~ Prevalence * Impact +
 (Prevalence * Impact | Participant) +
 (Prevalence * Impact | Item)


MCMC diagnostics indicated sufficient mixing of the chains, sufficiently high bulk and tail effective sample size values, and an $\hat{R}$ convergence diagnostic of 1.0 or below for all parameters.

**Figure 8:**
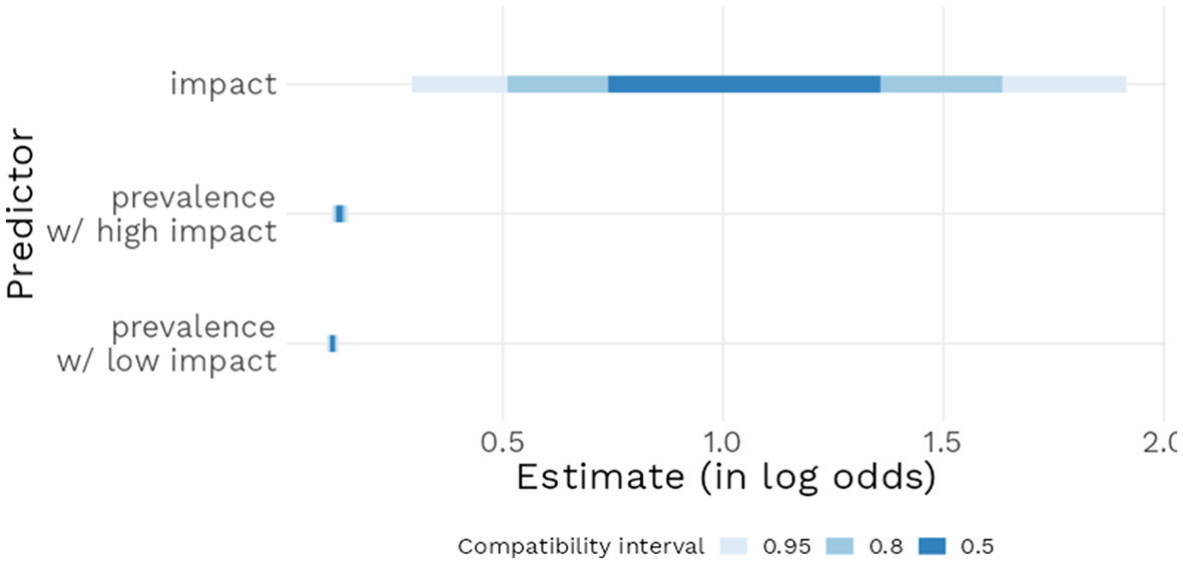
Posterior distributions of main model parameters with 95% compatibility interval.

**Figure 9:**
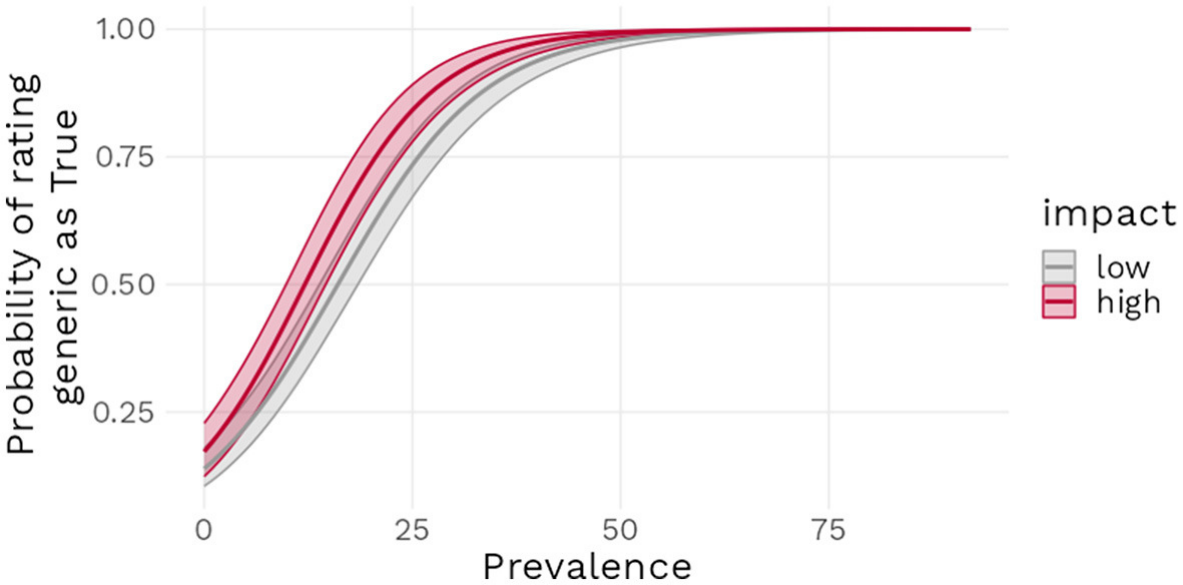
Predicted probability of truth ratings as a function of prevalence and impact condition with 95% compatibility interval.

Model 3 revealed a positive estimate [*b* = 3.86, 95% CI (3.29 : 4.46)] for the intercept, indicating that generics in the Low impact condition with a prevalence of 50% had a 97.9% mean estimated probability of being endorsed by participants. When a generic was in the High impact condition instead of Low, the model estimated a *b* = 1.06 log odds [95% CI (0.31 : 1.94)] increase in the probability of that generic being endorsed. Each percent increase in prevalence led to a *b* = 0.11 log odds [95% CI (0.10 : 0.13)] increase in the probability of endorsement and finally, the interaction term was estimated as a *b* = 0.02 log odds, [95% CI (0.00 : 0.04)), suggesting a rather weak effect, compatible with values close to zero (see Figure 8). Figure 9 further underlines these results: the predicted probability of a generic being endorsed increases sharply on the first 25% of the prevalence range, with low impact generics being less likely to be endorsed, and then reaches ceiling approval around 40% prevalence.

We cross-validated Model 3 by comparing it with four restricted models (see Table 3): a model that included both predictors but no interaction term between them (Model IP), a model that only included prevalence (Model P), a model that only included impact (Model I) and finally a null model (Model 0). The difference in ELPD between Model 3 and Models IP and P was not meaningfully larger than their respective standard errors, suggesting that including impact as a predictor did not meaningfully improve the predictions of Model 3. Meanwhile, the difference in ELPD between Model 3 and Models I and 0 were multiple times larger than the corresponding standard errors, indicating that a meaningfully superior predictive performance could be expected from Model 3 compared to the other two models.

**Table 3:** Cross-validation comparisons of the models.

**Model**	**Δ_ELPD_**	**(SE)**
3	**0.0**	**0.0**
IP	−2.2	1.8
P	−9.8	4.1
I	−984.8	32.3
0	−984.9	32.3

For each cross-validation comparison, the Δ_ELPD_ is the difference in ELPD between each model and the model with the largest ELPD (indicated in bold face). The Δ_ELPD_ for the best model is always 0 given that it is the difference between that model and itself. SE corresponds to the standard error of the difference in ELPD between two models and indicates the amount of uncertainty present in the comparison. The Δ_ELPD_ must be several times larger than the SE for the comparison to indicate meaningful differences in predictive performance between two models.

### 4.3 Discussion

Model 3 found further support for H1 and H3: increasing prevalence lead to a higher probability of generic endorsement and increasing the impact value of a generic did as well. Interestingly, and this prolongs our discussion on the *effect size* of impact, the estimated effect of impact at low prevalence levels was again not so strong that it would lead to a probability of endorsement above 50% and the effect of Impact was approximately equivalent as large as an increase in prevalence of almost 10%. The stability of our results across stimuli and dependent variable types points toward the claim that impact value plays a role in the meaning of generics but that this role is smaller than was previously assumed.

Before further elaborating on this claim, however, we need to turn to another concern that was raised in the Discussion of Experiment 2: according to the results from Cella et al. (2022), we might be overestimating the role of impact instead, as in the Truth-Conditions task of their Study 3a, the property valence predictor (or impact predictor, under our terminology) was not found to be statistically significant. To investigate this possibility, we began by reanalyzing their data with our own analytical approach. We chose to reanalyze the results from Cella et al. (2022) for three reasons: first, so they would be more directly comparable with our results; second, because we were concerned that the partly between-subject design structure of their experiment and the complexity of their model structure would not allow for proper model convergence under a frequentist framework, an issue that is not shared by a Bayesian statistical approach; third, in their Supplementary materials the authors reported excluding a portion of the recruited participants because of a difference in their “pattern of responses”, a difference that was due in part to the fact that the responses from the excluded sample revealed an effect of property valence. This seemed to us questionable grounds for exclusion (for more details on the reanalysis of Cella et al., 2022's data, see the Appendix). We used a Bayesian version of the same logistic mixed-effects regression model as the authors to predict the probability of a generic being endorsed, with Prevalence centered on 50% and the same priors as those used for Model 3 and included the full sample of recruited participants. We found a positive effect of Prevalence [*b* = 0.10, 95% CI (0.09 : 0.11)] and of Property valence [*b* = 0.47, 95% CI (0.16 : 0.77)], with the estimate for Property valence being, with this data, four to five times larger than the estimate for Prevalence. These findings offer therefore additional support for H1 and H3 and lay to rest the concern that we might be erroneously detecting an effect of impact; rather, impact value has an estimated effect on the probability of endorsement that is a few times larger than the effect of prevalence, while remaining smaller than expected by our proposed theory and the classical examples it attempts to account for.

## 5 Descriptive survey

**Figure 10:**
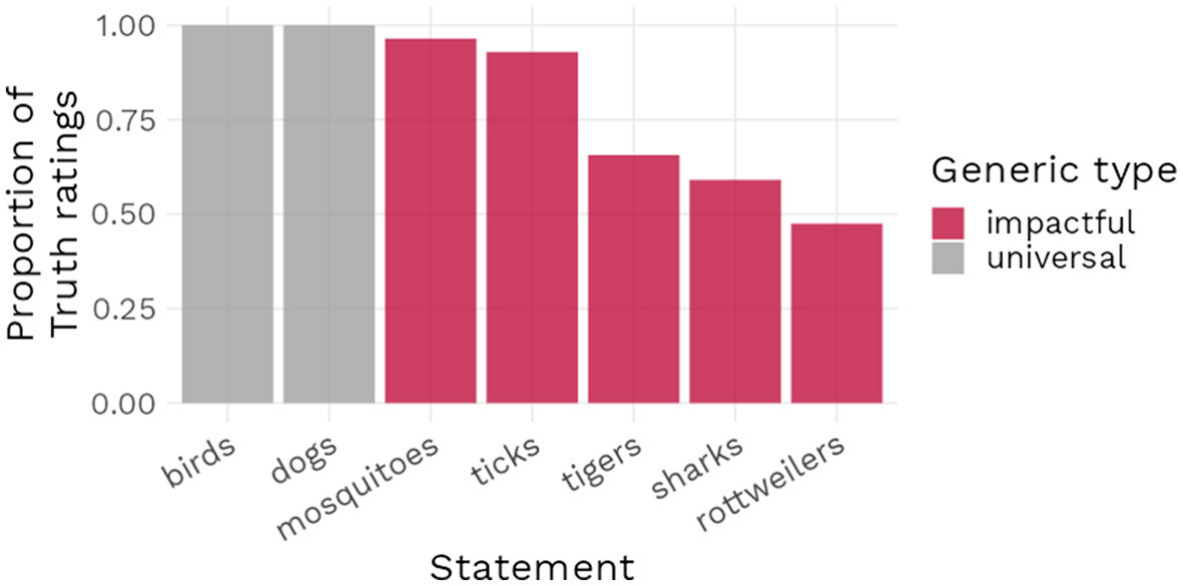
Proportion of truth ratings for universal generics and for common generics with high impact value and low prevalence.

As noted above, one constant of the findings presented in this paper is that increasing the negative impact of a statement did not generally lead to the positive endorsement rates for generics with low prevalence that our theory predicts. Indeed, our theory was developed in part to account for the statements with negative impact that have been identified in the literature as being felicitous. On the basis of those examples, we expected high endorsement rates for negative impact statements, which would have matched the intuitions of formal semanticists. Given that our results as exemplified in Figure 10 suggest instead a divergence between the endorsement rates for the stimuli used in our experiments and the endorsement rates implied by the theoretical literature, we can formulate two possible explanations.

A first possible explanation is that our theory, and therefore the highly controlled experiments we conducted, is not able to account for the totality of what makes a statement likely to be (highly) endorsed: we have found prevalence, contrast prevalence and impact to play a role in predicting assertability or endorsement, but more factors may need to be included in order to fully account for the endorsement rates of negative impact generic statements in everyday life. These factors might be, for instance, the personal relevance of the statement or the intensity of how information regarding the generic statement has been experienced or communicated. A second possible explanation is that the endorsement rates suggested by the theoretical literature finding negative impact generics to be felicitous might not reflect the actual endorsement rates of those generics by laypeople.

We chose to investigate more fully the latter explanation and conducted a simple descriptive survey, requesting participants to rate as being true or false a series of generic statements commonly used in the literature. Our sample of items included two uncontroversial generic statements (“Birds fly” and “Dogs bark”) with a high prevalence rate and no negative impact, five generic statements with a negative feature and a low prevalence rate (e.g., “Sharks attack bathers”) and one false generic statement (“Flamingos are green”), which served as an attention check. We collected responses from 199 participants (98 women, 98 men; M_age_ = 42.23, SD_age_ = 13.06), with none of them failing our attention check. We found that although almost all statements were more likely to be endorsed than not, there were notable variations in the levels of this endorsement, with the uncontroversial statements being accepted by 100% of participants while the statements with a negative impact were endorsed at levels varying from 96 to 47%. The results from this survey suggest that lay people's endorsement of common generics with low prevalence and negative impact value is far from unanimous. This in turn implies that, even if one chooses to retain impact value as an explanatory factor, it would be unrealistic to expect it to have a large effect in most experimental settings.

## 6 General discussion and conclusion

The observation that generics about negative impact features are likely to be endorsed despite their low prevalence levels seems easy to connect to why negative stereotypes are formed. At first, their emergence seems rational: protecting ourselves and others from the costly effects of negative, even if unlikely, features would be a valuable skill in an already complex, challenging world. On the other hand, the limitations of such generalizations also seem obvious: if only a few striking examples suffice to form the generalization, those generic beliefs overgeneralize by assuming that the negative unlikely features apply to the majority (or totality) of instances of a kind, leading to an overly cautious and, in the social realm, often discriminatory approach to the world and others.

Through three preregistered experiments as well as a reanalysis of data from Cella et al. (2022), we found further support in this paper for the proposal that the impact value of a feature influences the likelihood that a generic statement will be endorsed. Next to confirming results from Kochari et al. (2020) showing that increases in prevalence and in relative contrast lead to higher endorsement rates we found that, compared to low impact generics, generics with a high impact feature were more likely to be endorsed, in particular at low prevalence levels. These findings are in line with previous results in the literature, but they still require to be specified. Most importantly, and reiterating results from Bian and Cimpian, 2021,^13^ we found that although high impact features increased the likelihood of generics being endorsed at low prevalence levels, they generally did not increase this likelihood *enough* to reflect how high impact low prevalence generics are commonly endorsed in common language. While this can suggest that theories of generics that include impact as a factor may require some further development, the results from our observational survey about negative impact generics that are commonly used as examples in the theoretical literature also indicate that the endorsement of those generics is less unanimous and uniform than was previously assumed. This raises further questions, in particular regarding why some high impact generics are less endorsed than others. Considering the example that we studied in the descriptive survey there seems to be a relation with the direct or indirect experience people might have observing individual members of the group displaying the relevant property. We have much more individual encounters with rottweilers than with tigers. And while we might have close encounters with ticks, we hardly ever know for an individual tick we observe whether it carries Lyme disease or not. This might lead people to hold different beliefs about the actual risks related to the different categories of animals. Further research needs to show whether this possible explanation is on the right track.

However, even if part of the examples of low prevalence generics turn out to be less endorsed as expected based on self-reported intuitions of linguists, there is still need to explain those examples, like moskitos, repeated here as (1), which are generally considered true.

(1) Mosquitoes carry the West Nile virus.

One possible explanation can be based on an approach defended by Sterken (2015a,b). According to her, low prevalence generics like (1) are actually false. People accept there generics by mistake. Making this idea more concrete, one could, for instance, propose that a majority approach to the acceptance of generic sentences is a correct description of the semantic meaning of generic sentences, but that people systematically overestimate the probability of mosquitoes to carry the West Nile virus, and this is what leads them to accept the sentence in (1). That overestimation might be caused, directly or indirectly, by the impact of the relevant feature. However, we fail to observe this effect of impact in our experiments because the experimental setting we use (and the same holds for Cimpian et al., 2010a, Bian and Cimpian, 2021 or Cella et al., 2022) does not replicate the process sufficiently that leads people to believe the generalization expressed in (1).^14^ This line of approach might be worth exploring more in the future. For instance, one could consider experiments that study the endorsement of low prevalence generics together with probing the actual probability people assign to the generalization that is expressed. One could also focus on attempting to convey feature prevalence in a less static way than fixed grids of icons or textual stimuli and attempt to convey information about prevalence and features in a way that reflects better how information is received nowadays, in particular with social media and online information sources. This line of approach—rejecting the truth of low prevalence generics—also rises some interesting conceptual questions regarding the status of semantic meaning and the way it relates to cognitive behavior of humans.

In light of the various similar studies on the effect of impact on the meaning of generic sentences we want also to highlight another important point for future research. We believe that we should work toward adopting methodological practices that will allow for easier comparison of effect sizes between studies, that will focus on analyzing the practical meaning of those effect sizes and that would go hand in hand with theoretical practices that formulate expectations regarding those effect sizes. To make the most out of our research results, we need to be able to compare and aggregate results from different studies in an efficient and effective way.

## Data availability statement

The datasets presented in this study can be found in online repositories. The names of the repository/repositories and accession number(s) can be found in the article/Supplementary material.

## Ethics statement

The studies involving humans were approved by UvA Ethische Commissies FNWI headed by Dr. Eric Sennema. The studies were conducted in accordance with the local legislation and institutional requirements. The participants provided their written informed consent to participate in this study.

## Author contributions

PM: Conceptualization, Data curation, Formal analysis, Investigation, Methodology, Software, Validation, Visualization, Writing – original draft, Writing – review & editing. RR: Conceptualization, Funding acquisition, Investigation, Methodology, Project administration, Resources, Supervision, Writing – original draft, Writing – review & editing. KS: Conceptualization, Funding acquisition, Methodology, Project administration, Resources, Supervision, Writing – original draft, Writing – review & editing.

## Footnotes

^1^Cohen takes the relevant probability function to express frequencies in the limit case. This can explain why generics — in contrast to standard quantifiers like “all” or “some” — seem to come with an unrestricted domain.

^2^We refer the reader to Kochari et al. (2020) for a more detailed demonstration and exposition of that formal proposal.

^3^Chatlosh et al. (1985) have shown that stronger emotions promote faster learning. This is relevant if acceptance of generic “*G*s are *f*” is related with how fast an association is learned between features *G* and *f*.

^4^Though, it should be mentioned that in their first experiments the effect of distinctiveness was not significant. This led the authors to replicate the experiment, reporting significant results the second time.

^5^However, the authors of Bian and Cimpian (2021) point out some limitations of this work.

^6^We applied preregistered exclusion criteria. We excluded participants who failed the attention check and comprehension questions: the seriousness question at the end of the survey and the attention check trial included in the truth conditions questionnaire. In the attention check in the truth conditions questionnaire, participants are told that an animal has a feature with 100% prevalence. We excluded any participants who give a False rating to the generic statement.

^7^Because we use different statistical tools a direct comparison is not possible.

^8^Cimpian et al., 2010a and Bian and Cimpian, 2021 ask participants to rate the truth value of generic sentences, while we ask for assertability ratings. We will come back to this difference in the discussion of experiment 2 and in experiment 3.

^9^We used the same exclusion criteria as in the first experiment.

^10^We thank Andrei Cimpian for raising both of these concerns after a presentation of our work at the 2021 Generics and Stereotypes in Language Workshop in Amsterdam.

^11^Preregistration materials for Experiment 3 can be found at https://osf.io/vrs84/?view_only=7fb38ee8eed746549ef6df113ca0509e.

^12^Model 3 deviated from our preregistered model in that the prior for the intercept was widened to normal (0, 1) and the prior for the standard deviation was defined as normal (0.25, 0.25) rather than exponential (1). This change was done to resolve a convergence issue with the model.

^13^We should mention here that this is not something the authors of Bian and Cimpian, 2021 observe in their discussion of the data.

^14^The process might be mediated by dynamics of associative learning or by how often people hear or see reports about mosquitoes as the carriers of the West Nile virus, both of which will be affected by impact.
