# Supplementary material for: The role of impact on the meaning of generic sentences
Source: Front Psychol. 2024 Sep 23;15:1363390. doi: 10.3389/fpsyg.2024.1363390 (PMC11457378; doi:10.3389/fpsyg.2024.1363390)
Supplement: Supplementary file 1 [file Data_Sheet_1.pdf]

Appendix of 'The role of impact on the meaning of generic sentences'

Patricia Mirabile, Robert van Rooij and Katrin Schulz*
Institute for Logic, Language and Computation (ILLC),
University of Amsterdam

A Discussion and analysis of Study 3a by Cella et al. (2022)

Study 3a in **?** investigates whether the 'generics asymmetry' effect (where the implied prevalence of generics is perceived as higher than the prevalence required to accept a generic as true) can be replicated and extended to social generics, and whether this asymmetry holds in the cases where the generic feature varies in valence (neutral vs dangerous). The study finds that the asymmetry holds in all four conditions and it also reports further evidence in favor of social generics being more likely to be accepted than animal generics at low prevalence levels (10 percent). One important take-away from this study, and from the paper in general is that domain (or maybe domain-knowledge) seems to modulate the effect of prevalence and that the effect of valence is either low or indirect.

A closer examination at Study 3a revealed to us two issues in the analysis of the data.

First, when the authors discuss (on p.11 of the SOM) the recruitment method for Study 3a, they report that they began recruitment with the requirement that participants have the "Masters" qualification on MTurk, but when recruitment slowed down, they elected to waive the requirement for the Masters qualification. However, when examining the data, they indicate that they observed "differences between the pattern of responses across samples", which led them to discard the sample of non-Masters and replace it with a new sample of Masters. To put this more clearly, the inclusion of the non-Masters sample was conditioned on whether analyzing their responses revealed results similar enough to those from the analysis of the initial sample of Masters. This approach raises the following concerns: (1) The first sample of Masters and the non-Masters sample correspond each to roughly half of the preregistered planned sample size. Assuming that that sample size was chosen because it would ensure the experiment had sufficient power for the analysis, conducting analyses on half-sized samples would consequently lead to under-powered analyses and therefore to unreliable results. (2) The grounds for rejecting the Non-Masters sample seems to rely on the assumption that the effects detected on the Masters sample are more reliable and of higher quality. The first part of that assumption has been weakened in the first point above. Regarding the second part of the assumption, we agree that

*The names of the authors occur in alphabetical order.

participants with the Masters qualification might provide better responses (because they have proven more attentive or more able to accomplish more complex tasks in the past), however, this does not allow for the inference that Masters participants are more representative of human reasoning patterns than non-Masters participants. (3) This data collection approach amounts to using statistical significance findings as a guide for the inclusion or exclusion of participants. In doing so, the study unduly stacks the deck in favor of one theory instead of another, since it includes sampled groups on the condition that they preserve the previously obtained results of statistical significance. (4) Finally, the practice of testing for statistically significant predictors on the different sub-samples increases the risk of false positives, in particular in the frequentist framework of the reported analyses.

One possible solution this problem is to include the complete sample in the analysis and we apply this solution to the logistic regression model reported in the paper for Study 3a. This model focuses on the Truth Conditions task of the experiment. We first attempted to replicate the analyses reported by the authors, using the same frequentist framework and the same sample of only Masters-qualified participants, but found that the model failed to converge, meaning that the frequentist model is unable to provide stable estimates for the parameters and that the reported results are unreliable (our second issue with the paper's original analysis). This same convergence issue appeared also with the complete collected sample, so we turned to a Bayesian statistical model instead.

We fit a Bayesian logistic mixed-effects model on the responses to the Truth Condition task, using the complete collected sample. This model predicted the probability that a generic statement would be endorsed by a participant, using as predictors the prevalence of the generic (ranging from 10 to 100 percent and then centered on 50 percent), the valence of the property (neutral or dangerous) and the domain of the generic (animals or people). It also included the same priors, where applicable, as those used for Model 3. We found a positive effect of prevalence (b = 0.09 log odds, 95 percent CI [0.08:0.10]), such that an increase in prevalence clearly increased the probability that a generic would be endorsed.

We also found a small but positive effect of property valence for the Animal domain, where, for a prevalence of 50 percent, a dangerous property was more likely (b = 0.43 log odds, 95 percent CI [0.15:0.72]) to be endorsed than a neutral property. For the People domain, however, for a prevalence of 50 percent, a generic with a dangerous property was not meaningfully more likely to be accepted than a neutral property (b = -0.05 log odds, 95 percent CI [-0.32:0.21]). Figure 1 represents the probability of a generic being endorsed as a function of the prevalence of that generic, depending on both domain and property valence.

These results are in favor of an effect of property valence on generics. In particular, in the case of the Truth conditions task, we find that participants are more likely to endorse a generic with a dangerous feature than with a neutral feature, but that this result only holds in the case of generics about animals and not about people. Interestingly, however, the figure above suggests that generics about people, regardless of their valence, seem to be endorsed at rates similar to those of generics about animals with a dangerous feature. One possible explanation for this result, therefore, could be that participants tend to be more likely to endorse generics about people and that this in turn overpowers the effect of valence. Figure 2 reveals how the probability of endorsement tends to be higher for generics about people, in particular in cases where the prevalence of the generic is lower than 30 percent.

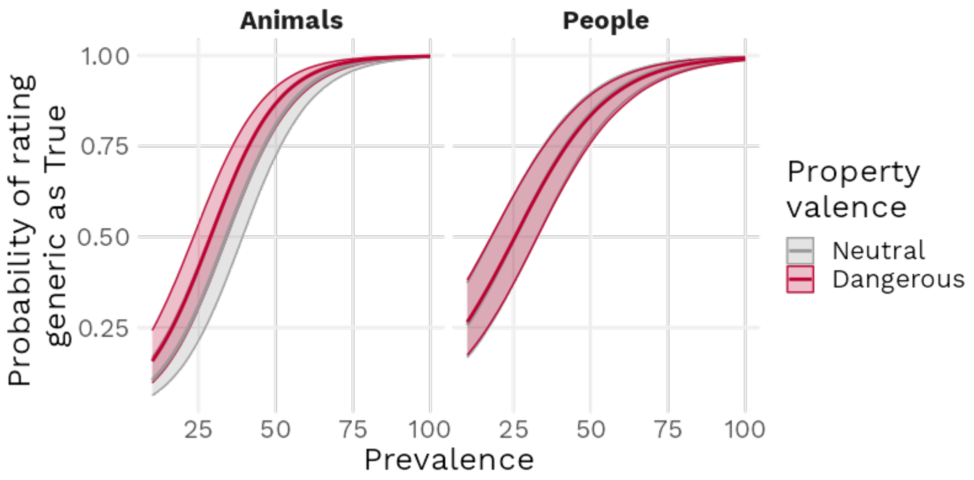

Figure 1: Predicted probability of truth ratings as a function of prevalence, property valence and domain.

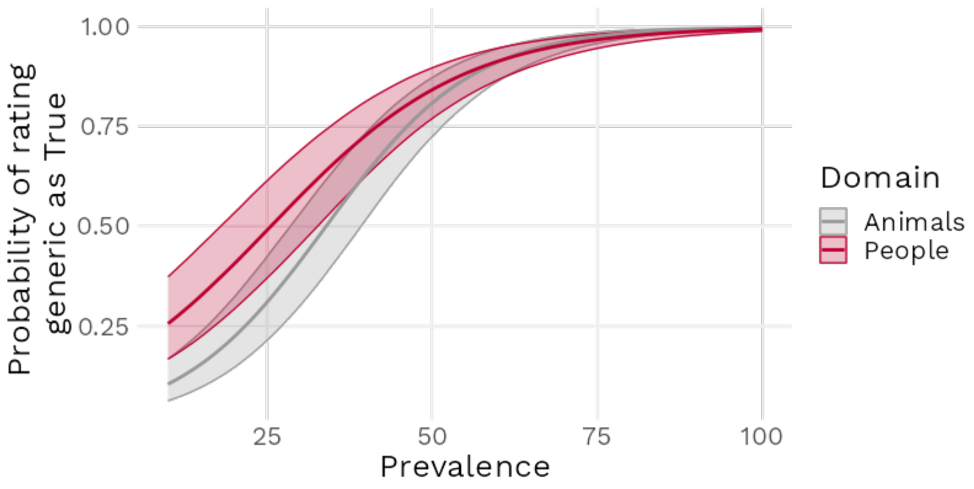

Figure 2: Predicted probability of truth ratings as a function of prevalence, property valence and domain.

B Example pictures of the different conditions of experiment 1

Below we provide example pictures from from the first experiment. Each contrast condition is exemplified once: one example for each condition with contrast, 80% vs. 12%, 92% vs. 20%, 12% vs. 20% and 80% vs. 90%, and one example for each condition without contrast, 12% and 12%, 20% and 20%, 80% and 80%, 92% and 92%. Half of the examples are pictures in the low impact condition, the other half are pictures from the high impact condition.

For all animals the experiment had a high impact and a low impact version.

Which animal the participant would see in which condition (low/high impact, level of contrast) was randomized. Each participant saw each animal only in one impact-condition.

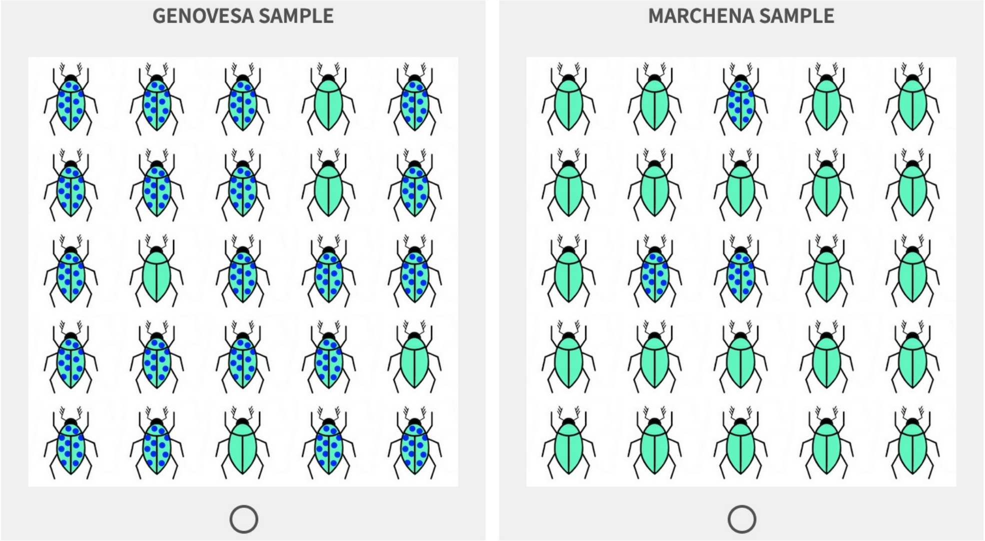

Figure 3: Stimuli example for the low impact condition with high contrast: 80% in the Genovesa sample vs. 12% in the Marchena sample. The generic sentence participants had to judge for this example was "[Genovesa/Marchena] beetles have blue dots."

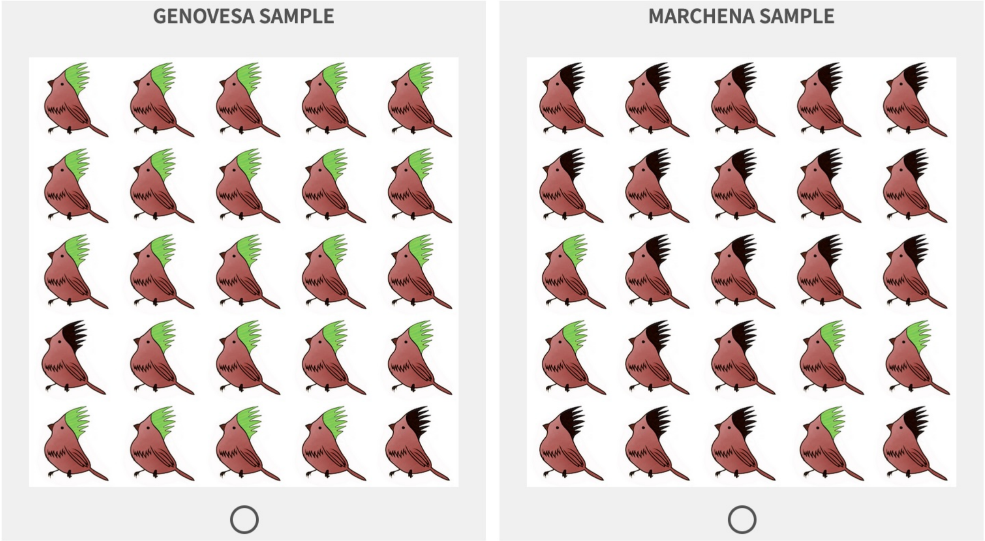

Figure 4: Stimuli example for the low impact condition with high contrast level: 92% in the Genovesa sample vs. 20% in the Marchena sample. The generic sentence participants had to judge for this example was "[Genovesa/Marchena] touracos have green crests."

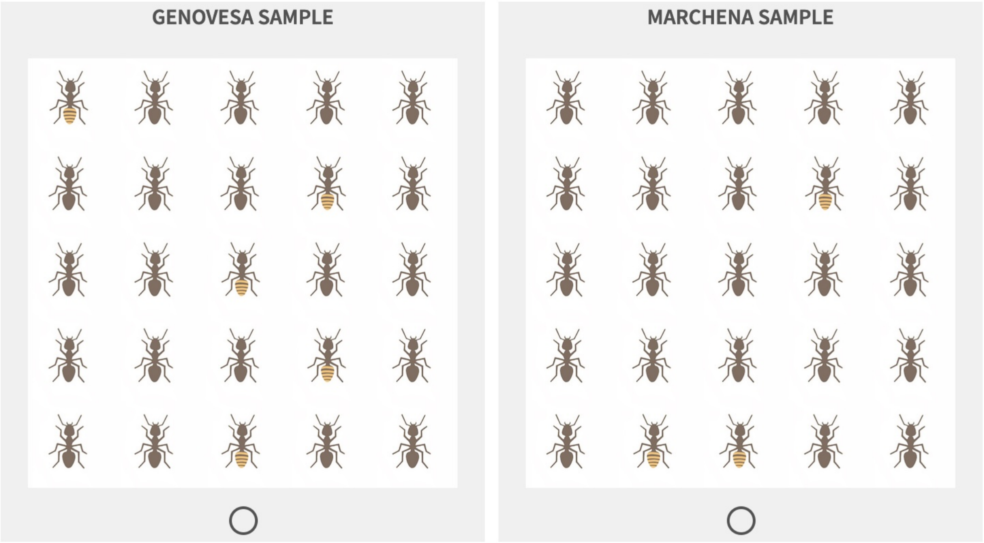

Figure 5: Stimuli example for the low impact condition with low contrast: 20% in the Genovesa sample vs. 12% in the Marchena sample. The generic sentence participants had to judge for this example was "[Genovesa/Marchena] ants have stripped bodies."

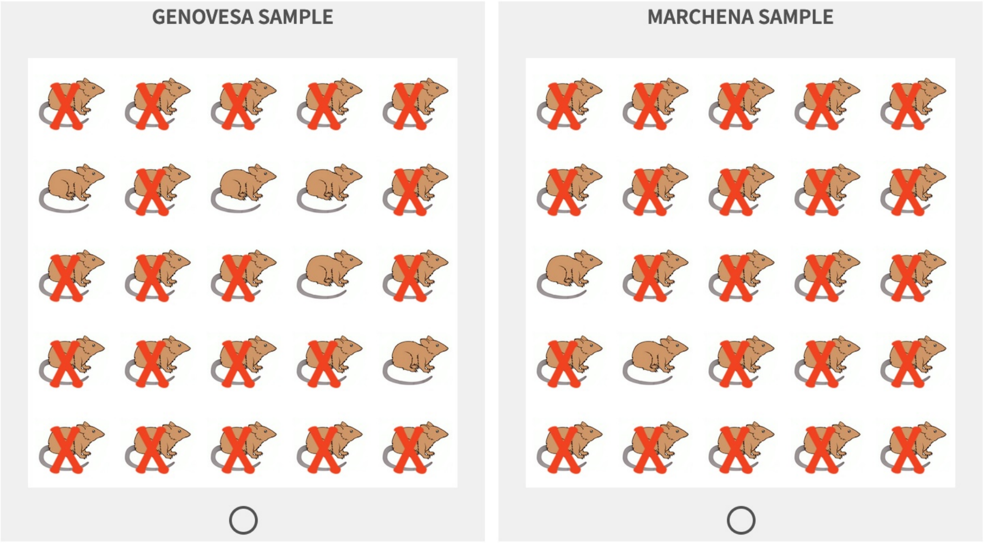

Figure 6: Stimuli example for the high impact condition with low contrast: 80% in the Genovesa sample vs. 92% in the Marchena sample. The generic sentence participants had to judge for this example was "[Genovesa/Marchena] mice are riddled with fleas."

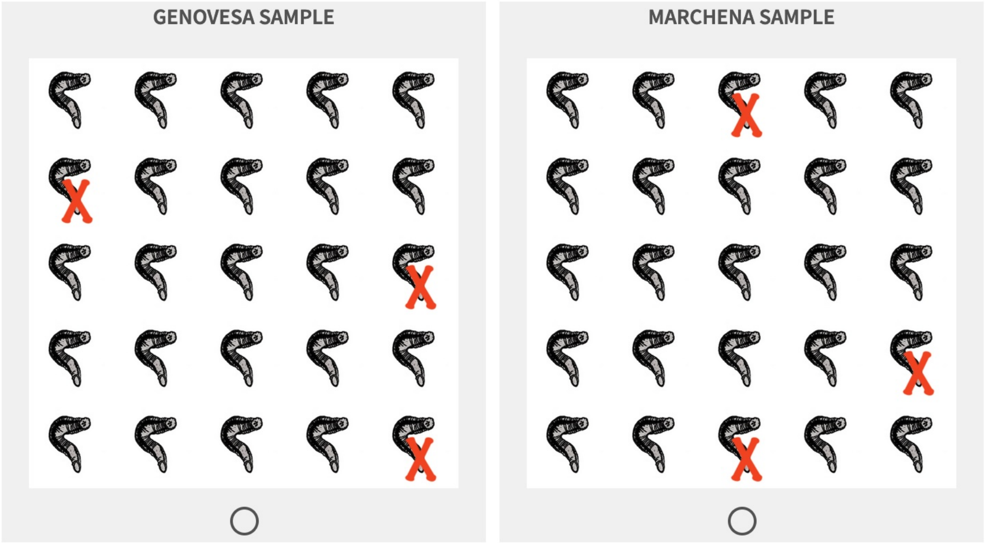

Figure 7: Stimuli example for the low impact condition with no contrast: 12% in the Genovesa sample and 12% in the Marchena sample. The generic sentence participants had to judge for this example was "[Genovesa/Marchena] leeches feed on human blood."

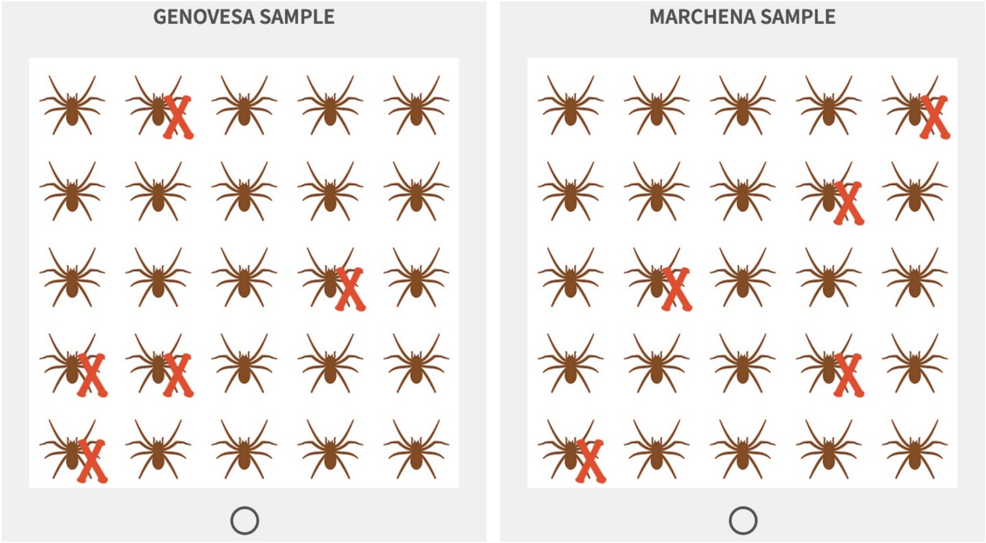

Figure 8: Stimuli example for the low impact condition with no contrast: 20% in the Genovesa sample and 20% in the Marchena sample. The generic sentence participants had to judge for this example was "[Genovesa/Marchena] spiders have a neurotoxic venom."

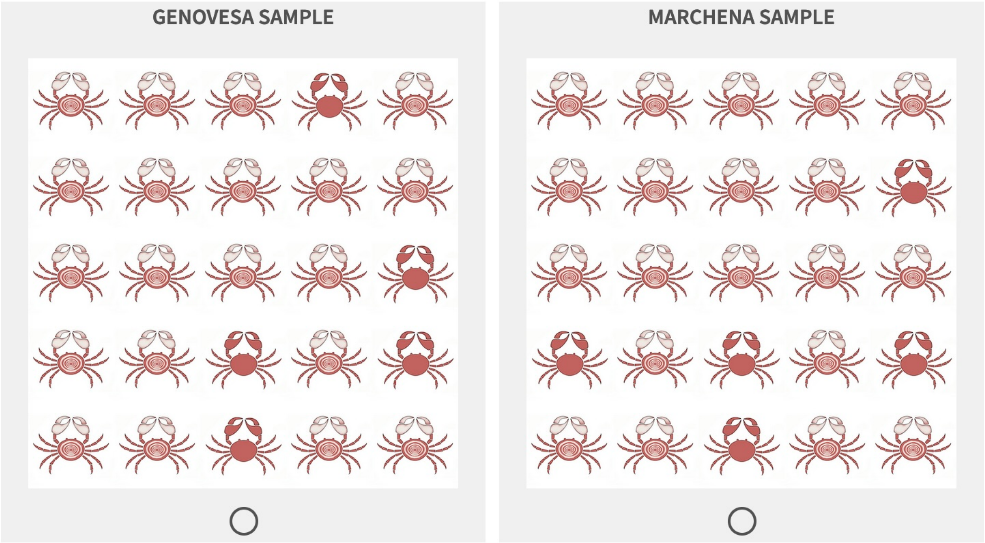

Figure 9: Stimuli example for the low impact condition with no contrast: 80% in the Genovesa sample and 80% in the Marchena sample. The generic sentence participants had to judge for this example was "[Genovesa/Marchena] crabs have white markings on their shells."

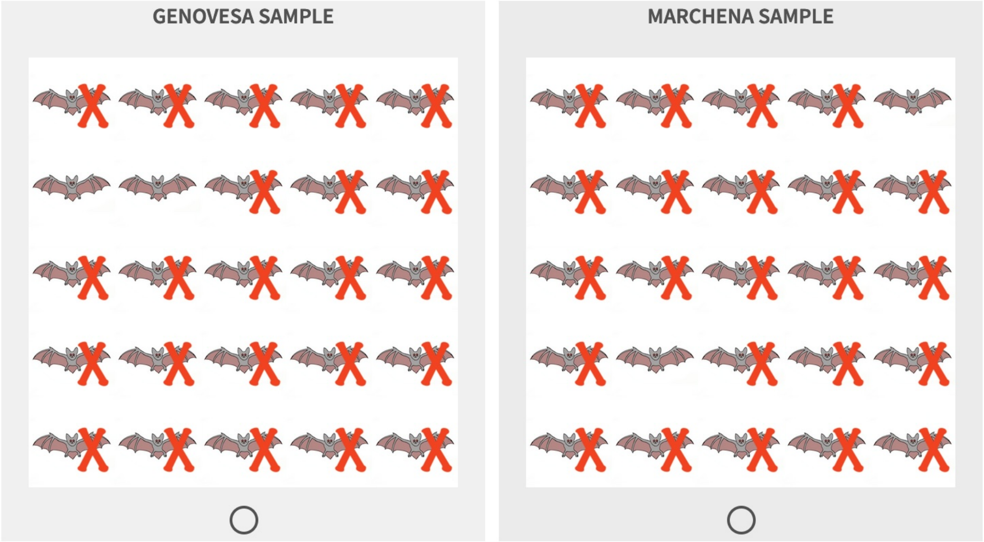

Figure 10: Stimuli example for the high impact condition with no contrast: 92% in the Genovesa sample and 92% in the Marchena sample. The generic sentence participants had to judge for this example was "[Genovesa/Marchena] bats are highly aggressive."
